# Roles of mitochondrial transcription factor A and microRNA-590-3p in the development of bladder cancer

**DOI:** 10.3892/ol.2013.1419

**Published:** 2013-06-21

**Authors:** MIAO MO, FENGHUA PENG, LU WANG, LONGKAI PENG, GONGBIN LAN, SHAOJIE YU

**Affiliations:** 1 Department of Urologic Organ Transplantation, The Second Xiangya Hospital of Central South University, Changsha, Hunan, P.R. China;; 2 Department of Otolaryngology, The Third Xiangya Hospital of Central South University, Changsha, Hunan, P.R. China

**Keywords:** mitochondrial transcription factor A, microRNA-590-3p, bladder cancer

## Abstract

Mitochondrial transcription factor A (TFAM) is required for mitochondrial DNA (mtDNA) replication and transcription. microRNAs (miRNAs) act as key factors in the regulation of gene expression. However, the roles of TFAM and certain miRNAs and their association in cancer development remain unclear. The present study reported that the expression of TFAM was significantly increased in bladder cancer, while the expression of miRNA-590-3p was downregulated. The luciferase assay showed that TFAM was the direct target of miRNA-590-3p. Furthermore, the forced overexpression of miRNA-590-3p significantly inhibited the proliferation, migration and colony-forming ability of 5637 cells, which was in contrast with the results from the forced overexpression of TFAM in the 5637 cells. Furthermore, cell proliferation- and migration-related genes, including phosphoinositide-3-kinase (PI3K), Akt, matrix metalloproteinase (MMP)-2 and MMP9, were significantly downregulated in the miRNA-590-3p-overex-pressing 5637 cells, but upregulated in the TFAM-overexpressing cells. In conclusion, the present study suggested that TFAM, a direct target of miRNA-590-3p, may play a significant role in the tumorigenesis of bladder cancer and thus may be a promising target for cancer therapeutics.

## Introduction

Mitochondrial transcription factor A (TFAM), a member of the high mobility group (HMG) box protein family, is required for mitochondrial DNA (mtDNA) replication and transcription. HMG proteins are often overexpressed in cancer cells and are involved in apoptosis (1). Furthermore, mitochondria are critical for cancer cell metabolism. Mitochondrial uncoupling regulates the metabolic shift to aerobic glycolysis, which is termed the Warburg Effect and is essential for the survival and proliferation of cancer cells (2,3). As a result, mitochondria are involved in the regulation of cancer cell survival and growth.

Bladder cancer is the most prevalent form of cancer among males. Urinary bladder cancers are detected in 3.71% of elderly males in the USA, indicating that the cancer is associated with aging and oxidative stress (4). The high expression of TFAM has been reported to be related with a poor prognosis in multiple malignant tumors, including ovarian cancer, endometrial carcinoma and colon cancer (5–7). TFAM is expressed not only in mitochondria, but also in nuclei (7). Furthermore, as well as being a transcription factor in mitochondria, TFAM also regulates the expression of nuclear genes. It has been reported that the number of mitochondria correlates with the growth rate of cancer cells and that the TFAM protein multimerizes and binds to mtDNA, suggesting that the TFAM levels may be increased in cancer cells and be associated with the malignant progression and proliferative activity (8). However, the roles of TFAM have not been fully identified in cancer cells.

microRNAs (miRNAs) are small, endogenous and non-coding RNAs that inhibit gene expression via the interaction with target sites in the 3′-untranslated regions (UTRs) of mRNA (9). miRNAs play significant roles in regulating multiple biological processes, including the pathogenesis of a variety of human cancers (10–12). miR-590-3p has been reported to be involved in mediating the expression of autoimmune genes and neuronal death (13,14). However, whether or not miR-590-3p is associated with malignant tumors remains unclear.

The present study aimed to elucidate the roles of TFAM and miR-590-3p and their association in bladder cancer cells.

## Materials and methods

### Cell culture

The human bladder carcinoma 5637 cell line (Institute of Cell Biology, Chinese Academy of Sciences, Shanghai, China) was cultured in RPMI-1640 medium with 10% FBS and 1% penicillin/streptomycin at 37°C, with 5% CO_2_.

### RNA extraction and quantitative (q)PCR analysis

RNA was extracted from the tissues or cell lines using TRIzol reagent (Invitrogen, Carlsbad, CA, USA), according to the manufacturer’s instructions. The RNA integrity was then assessed. A TaqMan qRT-PCR miRNA assay (Applied Biosystem, Carlsbad, CA, USA) was performed to detect the mature miR-590-3p expression levels. The relative expression of mature miR-590-3p levels normalized to U6 endogenous control was determined using the 2^−ΔΔCt^ method. Each measurement was performed in triplicate. To detect the target genes (mRNA expression), 1 *μ*g total RNA was reverse transcribed to cDNA using SuperScript™ III First-Strand Synthesis SuperMix (Invitrogen). For TFAM expression, SYBR Green qPCR Mix (Toyobo, Osaka, Japan) was used. SYBR green q-PCR was performed using the Bio-Rad iQ5 PCR detection system (Bio-Rad, Hercules, CA, USA) with the following gene-specific primers: forward, 5′-AAAGATTCCAAGAAGCTAAGGGTG-3′ and reverse, 5′-CCTAACTGGTTTCCTGTGCCTA-3′.

### Western blotting

The cells were solubilized in cold RIPA lysis buffer and then separated with 10% SDS-PAGE. Following this, the proteins were transferred to PVDF membranes. The membranes were blocked in 5% skimmed dried milk in PBS for 3 h and then incubated overnight with primary antibodies for TFAM (rabbit polyclonal), Akt (rabbit polyclonal), p-Akt (rabbit polyclonal), matrix metalloproteinase (MMP)-2 (mouse monoclonal), MMP-9 (rabbit polyclonal) (Abcam, San Francisco, CA, USA), phosphatidylinositol-3-kinase (PI3K; rabbit polyclonal), p-PI3K (rabbit polyclonal) and β-actin (mouse monoclonal) (Abzoom Biolabs, Dallas, TX, USA). Subsequent to being incubated with goat polyclonal anti-rabbit and goat anti-mouse secondary antibodies (Abcam), the immune complexes were detected using the enhanced chemiluminescence (ECL) method. The results were visualized by autoradiography using preflashed Kodak XAR film (Kodak, Tokyo, Japan).

### MTT assays

The cells were plated at a density of 5,000 cells per well. MTT (Promega, Madison, WI, USA) was added to the medium at a final concentration of 0.5 *μ*g/ml. The cells were then incubated at 37°C with 5% CO_2_ for 3 h. The medium was removed and 100 *μ*l DMSO was added into each well. The plate was gently rotated on an orbital shaker for 10 min to completely dissolve the precipitation. The absorbance was detected at 570 nm with a microplate reader (Bio-Rad).

### Dual Luciferase reporter assays

To generate the reporter vectors bearing miRNA-binding sites, a normal and mutated 3′-UTR of TFAM was subcloned using PCR-based methods. The constructs were inserted into the multiple cloning sites downstream of the luciferase gene in the psiCHECK-2 lucif-erase miRNA expression reporter vector.

For the luciferase assay, 10^5^ cells were cultured to ∼70–80% conf luence in 24-well plates and cotransfected with psiCHECK-2-TFAM-3′-UTR or psiCHECK2-mut-TFAM-3′-UTR vector plus 50 nM miR-590-3p or 100 mM miR-590-3p inhibitor using Lipofectamine 2000 (Invitrogen), according to the manufacturer’s instructions. The cells were incubated with transfection reagent/DNA complex for 5 h and refreshed with fresh medium containing 10% FBS. At 48 h post-transfection, firefly and renilla luciferase activities were evaluated using the dual-luciferase reporter assay system (Promega) and the renilla luciferase activity was normalized to firefly luciferase activity.

### Plasmid construction and transfection

TFAM, miR-590-3p and scramble miRNA [miR-SCR; negative control (NC)] plasmids were obtained from Auragene Bio (Changsha, China). The retroviral supernatants were prepared using Eco-Phoenix packaging cells and the 5673 cells were transduced using 20 mg/ml polybrene over 48 h.

### Colony formation assay

The colony formation rate was measured by a plate colony formation assay. In total, ∼200 cells were added to each well of a 6-well plate. The plates were incubated at 37°C for 14 days and gently washed and stained with crystal violet. Viable colonies that contained at least 50 cells were counted.

### Flow cytometric analyses

For the apoptosis analysis, 2×10^5^ cells were collected, washed twice with PBS and resuspended in 400 *μ*l 1X binding buffer. According to the manufacturer’s instructions, 5 *μ*l Annexin V-FITC and propidium iodide (PI) solution was then added. The samples were incubated for 15 min at room temperature and analyzed using flow cytometry (FACSCalibur; Beckman Coulter, High Wycombe, UK).

For the cell cycle analysis, the cells were collected in 1X PBS and resuspended in 70% ethanol to fix overnight at −20°C. The cells were pelleted, washed twice in 3% BSA in 1X PBS and pelleted again. The cells were resuspended and incubated for 30 min at room temperature in a PI staining buffer containing 3% BSA, 40 *μ*g/ml PI and 0.2 mg/ml RNase in 1X PBS. DNA content analyses were carried out using flow cytometry (FACSCalibur; Beckman Coulter).

### Transwell

For all the groups, migration was measured in 24-well Transwell chambers (Chemicon, Rosemont, IL, USA). Following a 24-h incubation period at 37°C, the migrated cells were stained with 0.04% trypan blue and counted with ×200 magnification.

### Statistical analysis

The data are shown as the mean ± SD of at least three determinations. The two-sided Student’s t-test was used to analyze the differences in the experiments. P<0.05 was considered to indicate a statistically significant difference. All statistical analyses were conducted using SPSS 17.0 (SPSS, Inc., Chicago, IL, USA).

## Results

### Protein expression of TFAM in the various tissues

**Figure 1.**
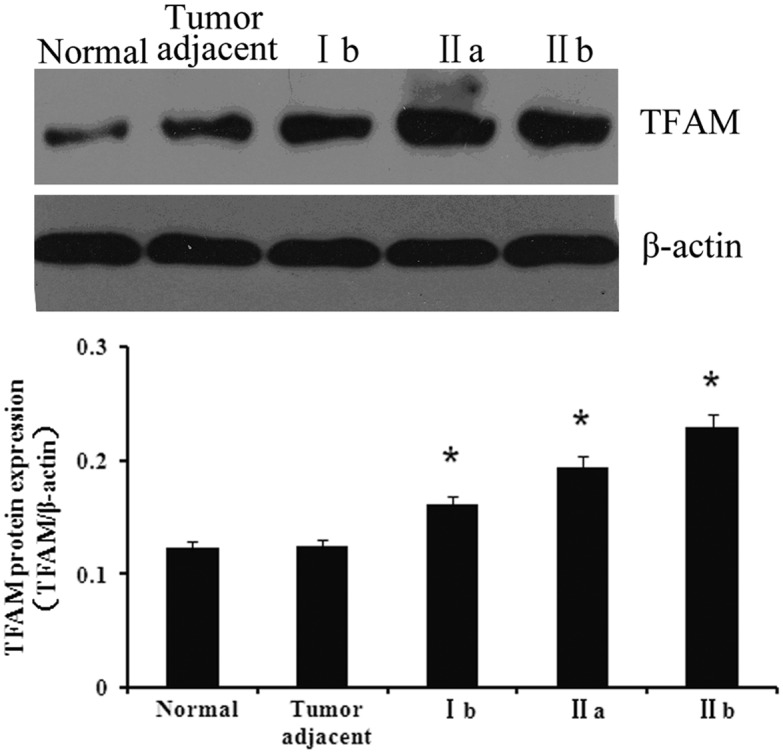
Protein expression of TFAM in bladder cancer. Western blot analysis was performed to determine the protein expression of TFAM in various grades of bladder cancers and in normal and tumor adjacent tissues. ^*^P<0.05 vs. normal. TFAM, mitochondrial transcription factor A.

To investigate the association between TFAM and bladder cancer, the expression of TFAM was first detected in normal, adjacent and bladder cancer tissues. As shown in Fig. 1, the expression of TFAM in the bladder cancer tissues was significantly higher compared with the normal and adjacent tissues (P<0.05). Furthermore, the tissues of the various cancer stages showed differing expression levels of TFAM. The expression of TFAM was at its highest in stage IIb tissues, whereas its expression in stage Ib tissues was the lowest.

### Expression of miR-590-3p in the various tissues

**Figure 2.**
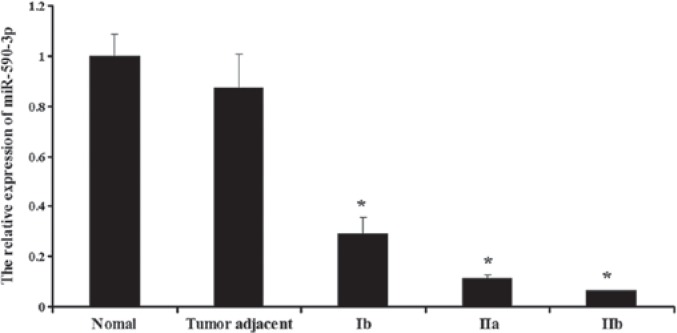
mRNA expression of miR-590-3p in bladder cancer. Quantitative (q)PCR was applied to examine the expression in various grades of bladder cancer as well as in normal and tumor adjacent tissues. ^*^P<0.05 vs. normal. miR, microRNA.

The expression of miR-590-3p was then determined in the normal, adjacent and bladder cancer tissues. As shown in Fig. 2, the bladder cancer tissues showed a lower expression of miR-590-3p compared with the normal and adjacent tissues (P<0.05). Furthermore, miR-590-3p displayed different expression levels in the various stages of bladder cancer. The expression in the stage Ib tissues was the highest, whereas the expression in the stage IIb tissues was the lowest (P<0.05). However, in the normal and adjacent tissues, the expression of miR-590-3p was not significantly different (P>0.05).

### Luciferase assay of miR-590-3p

**Figure 3.**
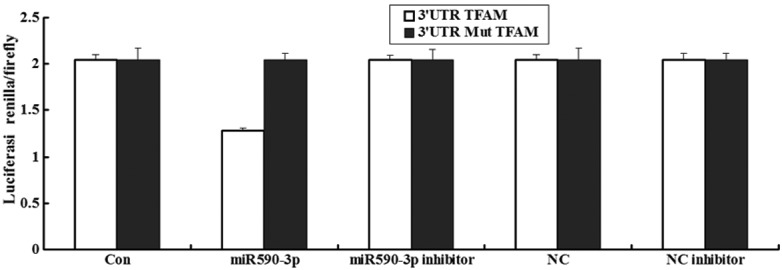
Luciferase assay of miR-590-3p. A luciferase assay was used to determine whether TFAM was the direct target of miR-590-3p. A normal and a mutated 3′-UTR of TFAM was subcloned into the psiCHECK™-2 luciferase miRNA expression reporter vector. PsiCHECK-2-TFAM-3′-UTR or psiCHECK-2-mut-TFAM-3′-UTR vector plus 50 nM miR-590-3p or 100 mM miR-590-3p inhibitor were cotransfected. Con, normal 5637 cells; NC, cells transfected with NC virus; NC inhibitor, cells transfected with NC inhibitor; miR, microRNA; TFAM, mitochondrial transcription factor A; UTR, untranslated region; qPCR, quantitative PCR; NC, negative control.

A luceriferase assay was performed to detect if TFAM was the direct target of miR-590-3p. The data showed that the renilla/firefly value of luciferase was significantly lower in the miR-590-3p treatment cells following transfection with the 3′UTR of the TFAM gene, while the renilla/firefly value of luciferase showed no difference following transfection with the mutated 3′UTR of TFAM compared with the control (Fig. 3). These data suggested that miR-590-3p may downregulate TFAM gene expression and that the 3′UTR of TFAM is the target of miR-590-3p.

### Detection of miR-590-3p and TFAM following transfection

**Figure 4.**
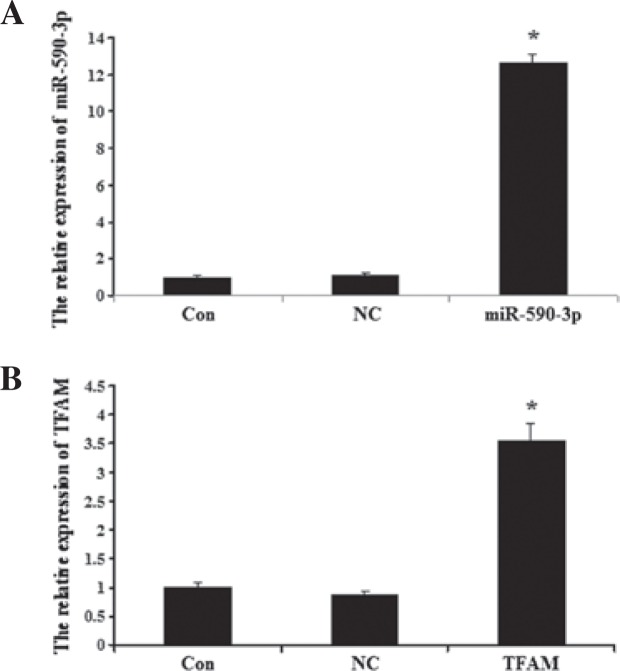
Detection of (A) miR-590-3p and (B) TFAM following transfection by qPCR. Con, normal 5637 cells; NC, cells transfected with NC virus; miR-590-3p, transfected with miR-590-3p lentiviral vectors; TFAM, transfected with mitochondrial transcription factor A (TFAM) lentiviral vectors. ^*^P<0.05 vs. control. miR, microRNA; qPCR, quantitative PCR; NC, negative control.

The data showed that the miR-590-3p expression level was significantly higher in the 5637 cells that were transfected with miR-590-3p lentiviral vectors compared with the controls (P<0.05; Fig. 4A). As shown in Fig. 4B, the expression of TFAM in the 5637 cells was upregulated following transection with the TFAM overexpression plasmid compared with the controls (P<0.05; Fig. 4B). No significant differences were observed between the expression of TFAM in the control cells and the cells that were transfected with the NC virus (P>0.05).

### Effect of miR-590-3p and TFAM overexpression on the proliferation of 5637 cells

**Figure 5.**
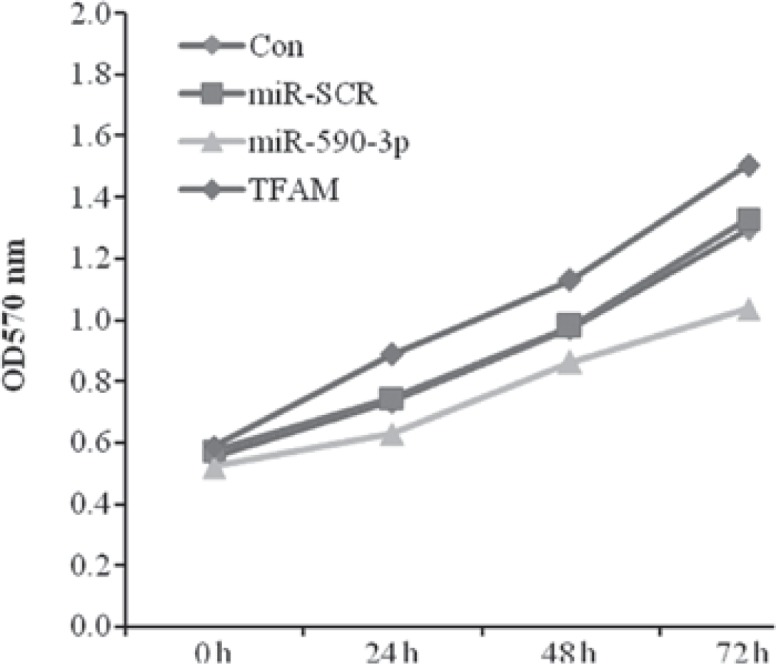
Effect of miR-590-3p and TFAM overexpression on the proliferation of 5637 cells. MTT was performed to determine the effect of the forced overexpression of miR-590-3p and TFAM on the proliferation of the 5637 cells. Con, normal 5637 cells; scramble miR (miR-SCR), 5637 cells that were transfected with miR-SCR lentiviral vectors; miR-590-3p, 5637 cells that were transfected with miR-590-3p lentiviral vectors; TFAM, 5637 cells that were transfected with mitochondrial transcription factor A (TFAM) lentiviral vectors; miR, microRNA.

As the expression level of miR-590-3p was lower in the bladder cancer tissues and the expression of TFAM was higher, the effect of the transfection of miR-590-3p and TFAM on 5637 cell proliferation was studied using MTT assays. The data showed significant cell growth inhibition in the miR-590-3p transfectant compared with the control from the 5367 cell lines. In contrast, the TFAM transfectant displayed a positive effect on cell growth (Fig. 5).

### Effect of miR-590-3p and TFAM overexpression on the cell cycle of 5637 cells

**Figure 6.**
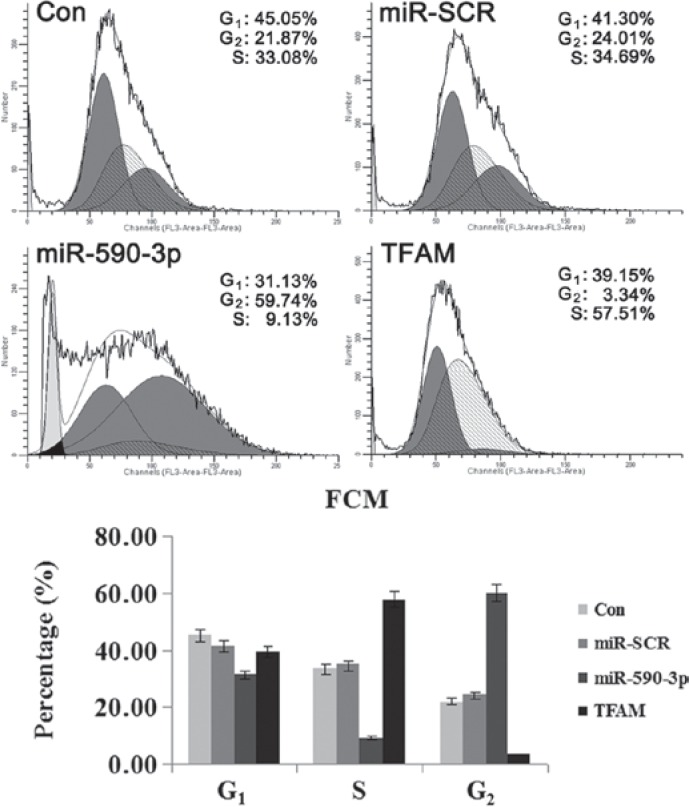
Effect of miR-590-3p and TFAM overexpression on the cell cycle of 5637 cells. A cell cycle assay was performed to determine the effect of the forced overexpression of miR-590-3p and TFAM on the cell cycle of 5637 cells. Con, normal 5637 cells; scramble miR (miR-SCR), 5637 cells transfected with miR-SCR lentiviral vectors; miR-590-3p, 5637 cells transfected with miR-590-3p lentiviral vectors; TFAM, 5637 cells transfected with mitochondrial transcription factor A (TFAM) lentiviral vectors; miR, microRNA.

As shown in Fig. 6, the cells that were transfected with the various vectors showed differences in the percentage of cells in the varying phases of the cell cycle. The cells that were transfected with miR-590-3p lentiviral vectors showed the highest percentage of cells in the G_2_ phase, suggesting that mitosis was blocked. For the cells transfected with TFAM over-expression vectors, the majority were in the G_1_ and S phases and only a few cells were in the G_2_ phase, indicating that cell division was active. These results indicated that TFAM promoted the mitosis of the 5637 cells, while miR-590-3p suppressed it.

### Effect of miR-590-3p and TFAM overexpression on the migration of 5637 cells

**Figure 7.**
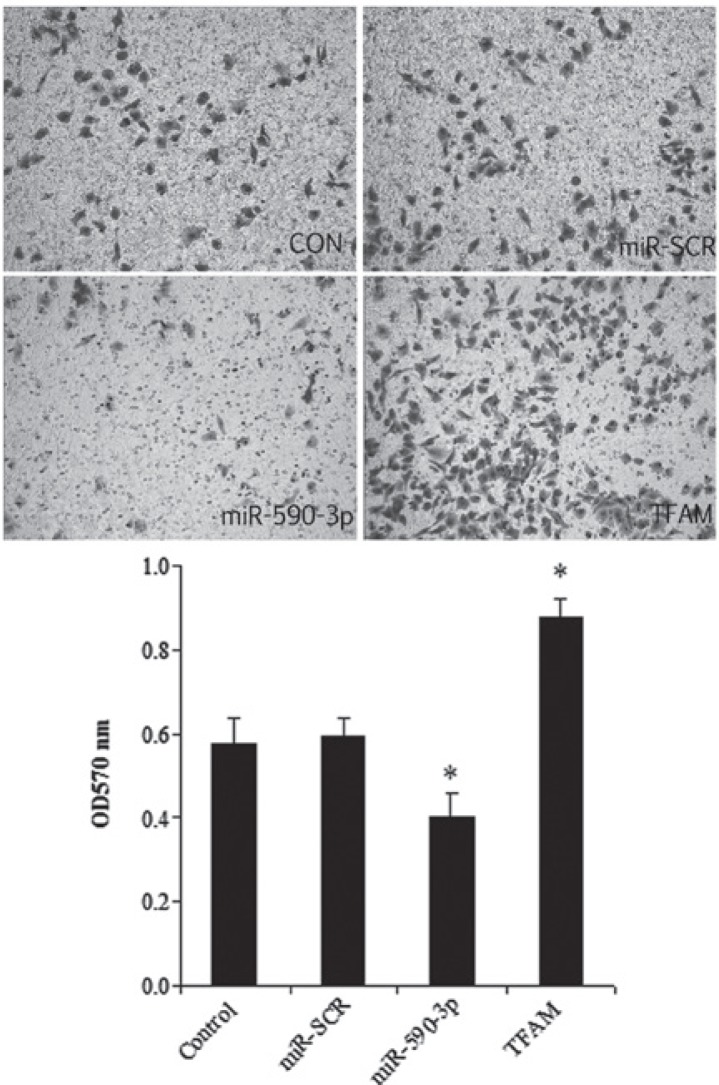
Effect of miR-590-3p and TFAM overexpression on the migration of 5637 cells. A Transwell assay was performed to determine the effect of the forced overexpression of miR-590-3p and TFAM on the migration of 5637 cells. Top panel, trypan blue staining; magnifiation, ×200. Con, normal 5637 cells; scramble miR (miR-SCR), 5637 cells transfected with miR-SCR lentiviral vectors; miR-590-3p, 5637 cells transfected with miR-590-3p lentiviral vectors; TFAM, 5637 cells transfected with mitochondrial transcription factor A (TFAM) lentiviral vectors. ^*^P<0.05 vs. control. miR, microRNA.

As shown in Fig. 7, the overexpression of TFAM significantly promoted cell migration, while the miR-590-3p groups had the lowest migration level among all the groups. In addition, a histological analysis revealed no significant difference between the control and miR-SCR groups.

### Effect of miR-590-3p and TFAM overexpression on the colony-formation efficiency of the 5637 cells

**Figure 8.**
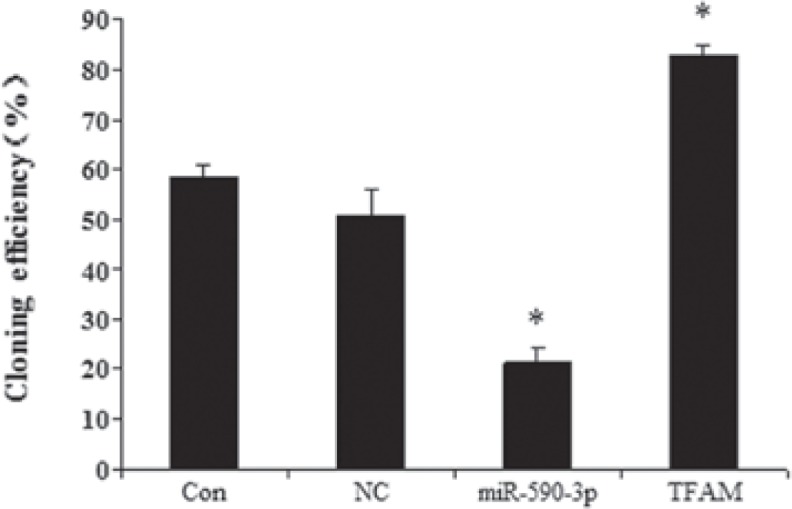
Effect of miR-590-3p and TFAM overexpression on the colony-formation efficiency of 5637 cells. A clone-formation assay was performed to determine the effect of forced overexpression of miR-590-3p and TFAM on colony-formation efficiency of 5637 cells. Con, normal 5637 cells; NC, cells transfected with NC virus; miR-590-3p, 5637 cells transfected with miR-590-3p lentiviral vectors; TFAM, 5637 cells transfected with TFAM lentiviral vectors. ^*^P<0.05 vs. control. miR. microRNA; TFAM, mitochondrial transcription factor A; NC, negative control.

The effects of TFAM and miR-590-3p on the colony-formation efficiency were examined in the human bladder 5637 cell line. Fig. 8 shows that the cells transfected with the different vectors had different colony-formation efficiencies. The cells that were transfected with the miR-590-3p lentiviral vectors showed the lowest colony-formation efficiency. The cells that were transfected with the TFAM overexpression vectors demonstrated the highest colony-formation efficiency. The control and miR-SCR groups showed no significant difference.

### Effect of miR-590-3p and TFAM on the expression of proliferation- and migration-related genes

**Figure 9.**
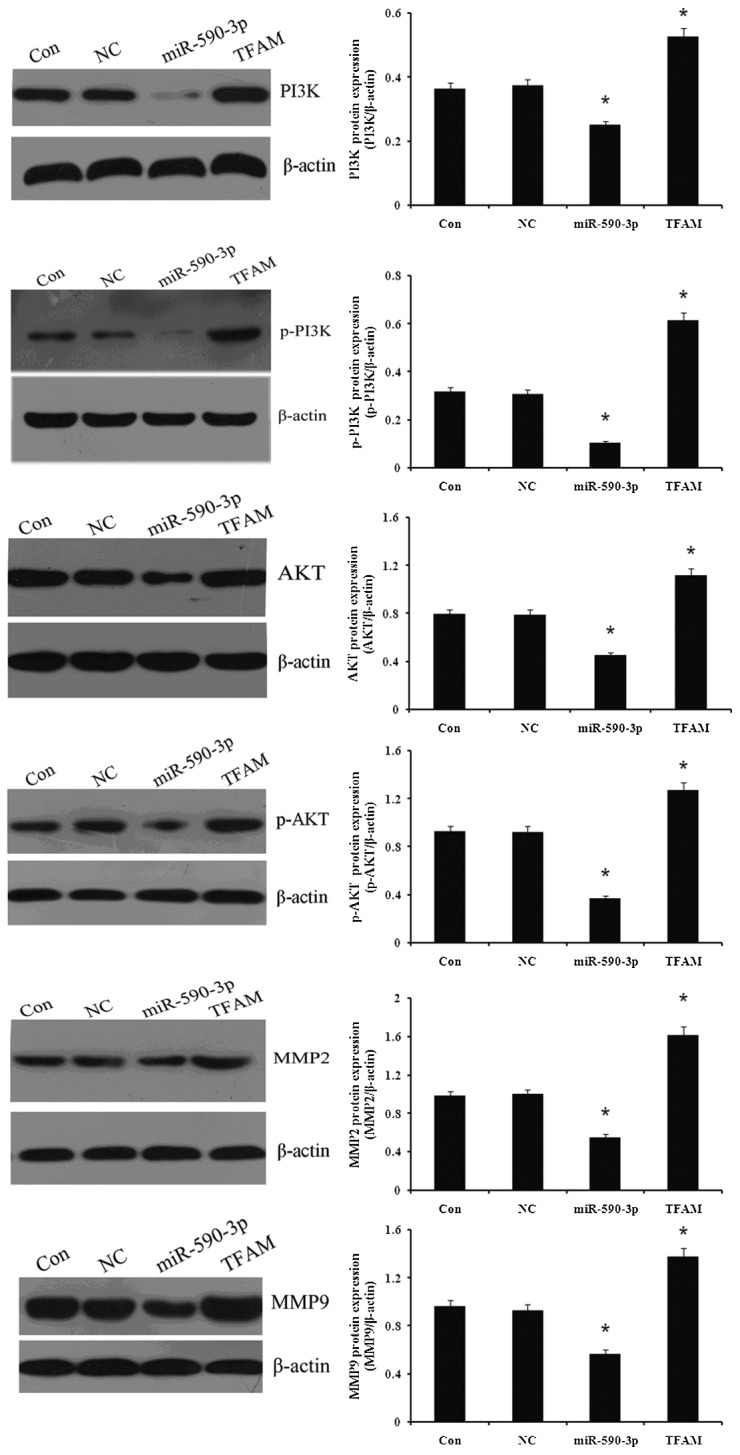
Effect of miR-590-3p and TFAM overexpression on proliferation- and migration-related genes expression in 5637 cells. Western blotting was used to determine the effect of miR-590-3p and TFAM overexpression on proliferation- and migration-related genes expression in 5637 cells. Protein expression levels of TFAM, PI3K, p-PI3K, Akt, p-Akt, MMP2 and MMP9 were examined and β-actin was used as an internal reference. Con, normal 5637 cells; NC, cells transfected with NC virus; miR-590-3p, 5637 cells transfected with miR-590-3p lentiviral vectors; TFAM, 5637 cells transfected with mitchondrial transcription factor A (TFAM) lentiviral vectors. ^*^P<0.05 vs. control. miR, microRNA; PI3K, phosphotidite-3-kinase; MMP, matrix metalloproteinase; NC, negative control.

The protein expression of certain proliferation- and migration-related genes was detected in the 5637 cells, which were transfected with the miR-590-3p or TFAM vectors, respectively. As demonstrated in Fig. 9, the western blot analysis indicated that the expression of PI3K, p-PI3K, Akt, p-Akt, MMP2 and MMP9 was decreased in the cells that were transfected with miR-590-3p lentiviral vectors, while the expression was increased in the cells that were transfected with TFAM overexpression vectors when compared with the controls (P<0.05). No significant difference in the gene expression was identified between the cells that were transfected with NC and the control cells. (P>0.05).

## Discussion

miRNAs represent a class of snRNAs that have central roles in gene silencing and function as part of large gene regulatory networks (15). In animals, miRNAs hybridize to partially complementary binding sites that are typically located in the 3-UTR of target mRNAs and repress their expression (16). Recently, miRNAs were identified to have significant roles in tumorigenesis (15). Previous studies have demonstrated that certain miRNAs are significantly deregulated in bladder cancer and may function as tumor suppressors (17,18). However, the role of miRNA-159-3p in the development of bladder cancer remains unknown. The present study reported that the expression of miRNA-159-3p was decreased in bladder cancer tissues compared with normal and adjacent tissues. Furthermore, the data showed that the higher the cancer grade, the lower the miRNA-159-3p expression in the bladder cancer tissues.

TFAM was first purified and cloned as a transcription factor for mtDNA. TFAM is able to enhance mtDNA transcription using mitochondrial RNA polymerase in a promoter-specific fashion (19). Since the replication of mammalian mtDNA is proposed to be coupled with transcription, TFAM is thought to be essential for the replication of mtDNA (20). It has been reported that TFAM may function to promote cell survival and proliferation. TFAM-null mice have been demonstrated to possess an embryonic lethal phenotype and exhibit apoptosis in heart cells (21). The knockdown of TFAM expression has been shown to induce p21-dependent G_1_ cell cycle arrest (22). Indeed, the present data showed that in bladder cancer, the expression of TFAM was significantly higher compared with the normal and adjacent tissues, indicating that TFAM may be associated with the growth of bladder cancer.

Notably, the expression of miRNA-159-3p and TFAM in cancer tissues was shown to be negatively correlated, which indicated that miRNA-159-3p may inhibit the expression of TFAM. The results of the luciferase assay demonstrated that miRNA-159-3p was able to directly downregulate TFAM expression, whereas the mutated miRNA-159-3p did not. The present data demonstrated that TFAM is the direct target of miRNA-159-3p. To the best of our knowledge, there has been no earlier study reporting the interaction between TFAM expression and these particular miRNAs. The cause of TFAM overexpression is unknown, but one possible mechanism is through the regulation by miRNAs. The loss of miRNA-159-3p, the endogenous TFAM inhibitor, may promote the aberrant expression of TFAM, contributing to the pathogenesis and progression of bladder cancer.

Furthermore, the present study investigated the molecular function of miR-590-3p and TFAM in the bladder cancer 5637 cell line. The MTT data showed that TFAM transfection significantly promoted cell proliferation in the 5637 cells, while miR-590-3p markedly decreased the level of cell proliferation. A previous study demonstrated that TFAM in the colorectal carcinoma cell line RKO carrying a TFAM truncating mutation suppressed cell proliferation and inhibited RKO cell-induced xenograft tumor growth (23). miR-590-3p suppressed the growth rate of the 5637 cells in the present study. The cell cycle results showed that the 5637 cells that were transfected with miR-590-3p had the highest percentage of cells in the G_1_ phase and the lowest percentage of cells in the S and G_2_ phases. For the 5637 cells that were transfected with TFAM overexpression vectors, the majority were in the S and G_2_ phases and only a few were in the G_1_ phase. To detect the molecular regulatory pathway of miR-590-3p and TFAM in the 5637 cells, western blotting was performed to determine the protein expression of PI3K, p-PI3K, Akt, p-Akt, MMP2 and MMP9 after the forced overexpression of miR-590-3p or TFAM. TFAM transfection significantly promoted PI3K, p-PI3K, Akt, p-Akt, MMP2 and MMP9 protein expression. In contrast, miR-590-3p showed significant deregulation of protein expression of PI3K, p-PI3K, Akt, p-Akt, MMP2 and MMP9. Metastasis is a complex and multi-step dispersion process of malignant tumor cells from the primary tumor site to a secondary site within the body. Therefore, the activation of the zinc-binding endopeptidases, the MMPs, is considered to play a crucial role in the process of cancer invasion and metastasis (24). MMPs are primarily regulated at the transcriptional level through AP-1 or NF-κB via mitogen-activated protein kinase (MAPK) or PI3K-Akt pathways, at post-transcriptional levels, at the protein level via their activators or inhibitors or at their cell surface localization (25–27). The overexpression of MMP-2 and MMP-9 in malignant tumors has been demonstrated to develop a vasculature by angiogenesis (28). In the present study, TFAM was shown to be able to promote this pathway. miR-590-3p was able to downregulate TFAM expression. Thus, the deregulatory effect of miR-590-3p on the pathway should be associated with TFAM.

In summary, the present study identified miR-590-3p and TFAM transfection expression patterns in bladder cancer. Using the luciferase assay, TFAM was identified as a target of miR-590-3p. The study was also extended into the molecular functions of miR-590-3p and TFAM. miR-590-3p was able to deregulate the metastasis pathway and may be used a new target for the therapy of bladder cancer.
